# Understanding neural development and diseases using CRISPR screens in human pluripotent stem cell-derived cultures

**DOI:** 10.3389/fcell.2023.1158373

**Published:** 2023-04-10

**Authors:** Mai Ahmed, Julien Muffat, Yun Li

**Affiliations:** ^1^ Program in Developmental and Stem Cell Biology, The Hospital for Sick Children, Toronto, ON, Canada; ^2^ Program in Neurosciences and Mental Health, The Hospital for Sick Children, Toronto, ON, Canada; ^3^ Department of Molecular Genetics, University of Toronto, Toronto, ON, Canada

**Keywords:** CRISPR, human pluripotent stem cell (hPSC), neurodevelopment, neurological disease, genetic screens, organoids

## Abstract

The brain is arguably the most complex part of the human body in form and function. Much remains unclear about the molecular mechanisms that regulate its normal and pathological physiology. This lack of knowledge largely stems from the inaccessible nature of the human brain, and the limitation of animal models. As a result, brain disorders are difficult to understand and even more difficult to treat. Recent advances in generating human pluripotent stem cells (hPSCs)-derived 2-dimensional (2D) and 3-dimensional (3D) neural cultures have provided an accessible system to model the human brain. Breakthroughs in gene editing technologies such as CRISPR/Cas9 further elevate the hPSCs into a genetically tractable experimental system. Powerful genetic screens, previously reserved for model organisms and transformed cell lines, can now be performed in human neural cells. Combined with the rapidly expanding single-cell genomics toolkit, these technological advances culminate to create an unprecedented opportunity to study the human brain using functional genomics. This review will summarize the current progress of applying CRISPR-based genetic screens in hPSCs-derived 2D neural cultures and 3D brain organoids. We will also evaluate the key technologies involved and discuss their related experimental considerations and future applications.

## Introduction

Model organisms-based genetic screens have long been favorite discovery tools for neuroscientists. Elegant forward genetic screens have brought unparalleled insights into how genes control neurodevelopment and functions, investigating flies that shake (Hotta and Benzer, 1972; Papazian et al., 1987), fish that cannot see (Brockerhoff et al., 1995) or mice that sleep too much (Funato et al., 2016), to name a few. In addition, human genetics research, either through linkage analysis or genome-wide association studies (GWAS), has contributed tremendously in linking disease phenotypes (such as autism or Alzheimer’s disease) with specific genome alterations. Conversely, reverse genetic screens examine the consequence of specific genetic alterations on biological processes. This approach has become increasingly popular with the advent of precise genome sequence information and tools for targeted editing. Efforts such as the Knockout Mouse Project have generated a targeted knockout mutant of every gene in the mouse genome (Austin et al., 2004). Excitingly, the convergence of targeted genetic modification and next-generation sequencing technologies has unleashed the potential of genome-wide reverse genetic screens. These screens are often carried out in cell lines and model organisms, and have allowed the large-scale, unbiased identification of the different genes and pathways that underlie development and disease (Gönczy et al., 2000; Berns et al., 2004; Neely et al., 2010; Shalem et al., 2014; Sin et al., 2014; Marceau et al., 2016). While earlier iterations of these screens in mammalian cells used gene silencing *via* RNA interference (Moffat et al., 2006; Boutros and Ahringer, 2008), recent studies have adopted the RNA-guided CRISPR (clustered regularly interspaced short palindrome repeats) associated nuclease Cas9 technology because of its superior precision, versatility, and ease of use (Koike-Yusa et al., 2014; Shalem et al., 2014; Wang et al., 2014). Of particular relevance to neurobiologists, CRISPR-based screens have been utilized for the high throughput systematic interrogation of genetic modifiers for phenotypes associated with diseases such as Alzheimer’s (Sanchez et al., 2021), Parkinson’s (Potting et al., 2018) and amyotrophic lateral sclerosis (ALS) (Chai et al., 2020). Although most of these studies relied on the use of cancer cell lines, which may limit the direct translation of the findings to the relevant diseases.

Recent advances in human pluripotent stem cells (hPSCs) technologies have provided a systematic platform for functional genomics studies using CRISPR in relevant, non-transformed human cell types (Tian et al., 2019; McTague et al., 2021; Dräger et al., 2022; Leng et al., 2022). hPSCs include embryonic stem cells derived from the human blastocysts (Thomson et al., 1998) and induced pluripotent stem cells (iPSCs) reprogrammed from somatic cells (Takahashi et al., 2007). To date, many studies have reported the successful differentiation of hPSCs into different neuronal cell types, including cortical excitatory (Cao et al., 2017), dopaminergic (Nolbrant et al., 2017), interneurons (Maroof et al., 2013), and motor neurons (Du et al., 2015) among others. The generation of non-neuronal cells of the brain such as microglia (Muffat et al., 2016; Abud et al., 2017; Pandya et al., 2017), astrocytes (Krencik and Zhang, 2011; Tcw et al., 2017), and oligodendrocytes (Douvaras et al., 2014; García-León et al., 2020) from hPSCs has also been established. These efficient 2D differentiation methods promise unlimited supplies of physiologically relevant neural cells for gene function studies. Furthermore, hPSCs can be differentiated into 3D self-organizing brain organoids, which recapitulate many aspects of the early fetal development such as cell migration and neural cytoarchitecture (Lancaster et al., 2013; Lancaster and Knoblich, 2014). More recently, brain assembloids were generated by integrating organoids representing different brain regions together (Bagley et al., 2017; Sloan et al., 2018), thereby offering a useful platform to model interactions between different brain regions and study neural circuit assembly.

**FIGURE 1:**
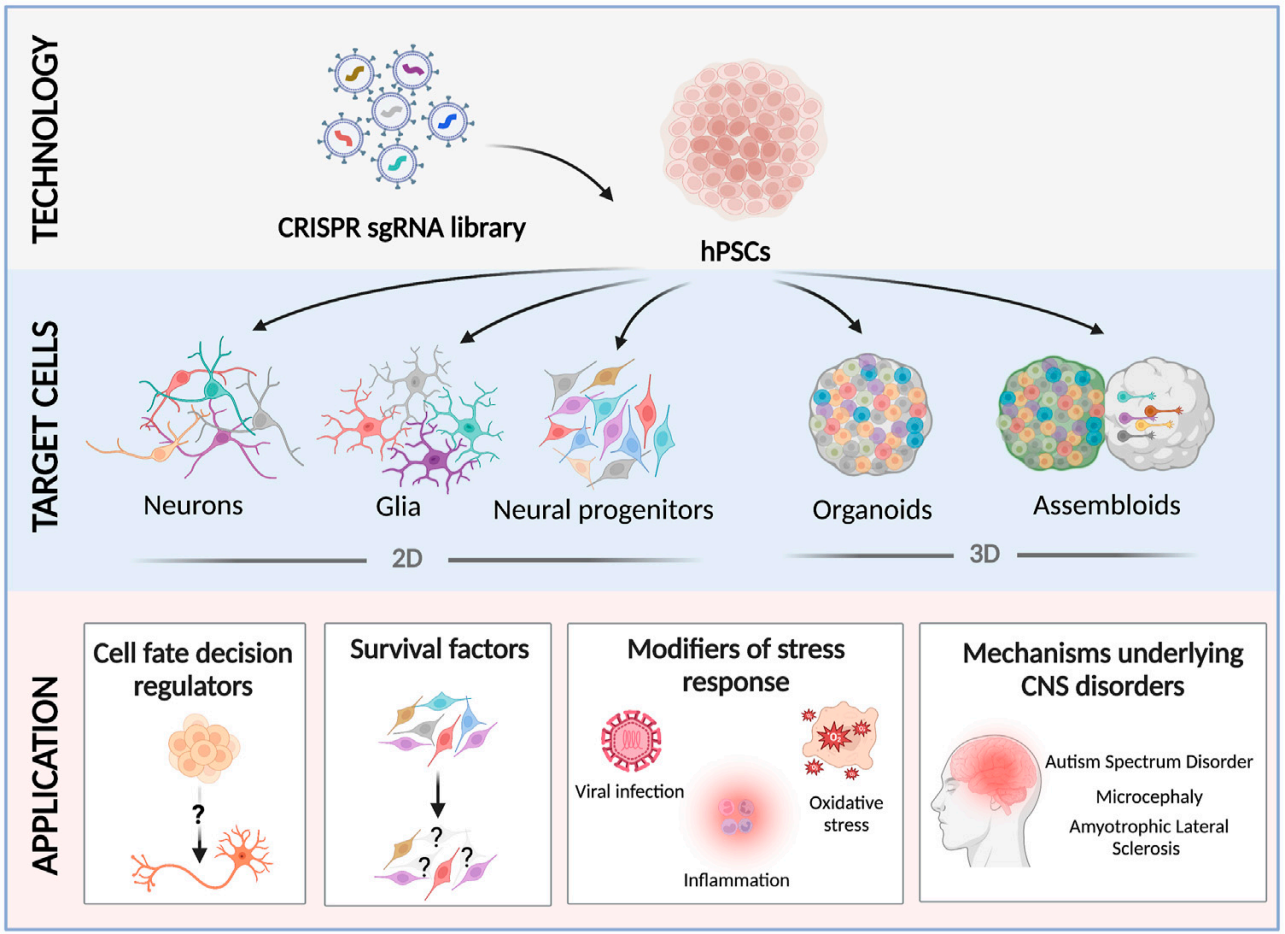
Recent applications of CRISPR screens using hPSC-derived neural cultures. hPSCs are transduced with a scalable pooled lentiviral sgRNA CRISPR library, and differentiated into 2D neural cultures (neural progenitor cells (Li et al., 2019), neurons (Tian et al., 2019; Tian et al., 2021), microglia (Dräger et al., 2022), or astrocytes (Leng et al., 2022)), or 3D brain organoids/assembloids (Esk et al., 2020; Fleck et al., 2022; Li et al., 2022; Meng et al., 2022). Screening neural cultures carrying different pooled CRISPR perturbations has revealed novel factors that regulate neurodevelopmental cell fates (Black et al., 2020; Fleck et al., 2022), essential genes (Tian et al., 2019; Tian et al., 2021), modifiers of cell survival under different challenges (Li et al., 2019; Tian et al., 2021; Leng et al., 2022), and the cellular and molecular mechanisms that underlie neurodevelopmental and degenerative disorders (Esk et al., 2020; Guo et al., 2022; Li et al., 2022; Meng et al., 2022).

Here we discuss recent studies that utilized these hPSC-derived 2D and 3D neural cultures in CRISPR-based genetic screens (Figure 1). Since excellent reports exist that extensively reviewed the technologies involved in CRISPR-based screens (Kampmann, 2018; Kampmann, 2020; Ford et al., 2019; Bock et al., 2022), we will briefly summarize their use to date in the context of studying neurological diseases, and focus the rest of the review on their recent application and future utility.

## Functional genomics using CRISPR-Cas9

### CRISPR technologies for genetic screens

CRISPR knockout (CRISPR-KO) is the most common strategy used in CRISPR-based genetic screens to date. It generates efficient and precise editing of the genome using a two-component system, the Cas9 protein and a synthetic single guide RNA (sgRNA) that targets Cas9 to specific genomic loci to introduce double-stranded DNA breaks (DSBs). DNA repair machinery is activated to repair those DSBs either by homology-directed repair or non-homologous end joining. In the absence of a repair template, non-homologous end joining will result in insertions-deletions that can introduce frame-shift mutations leading to loss of function (Sternberg et al., 2014). Genome-wide CRISPR-KO screens have been successfully carried out using lentiviral sgRNA libraries in different human cell lines to identify genetic drivers of tumorigenicity, pluripotency, immune response, and host-virus interactions (Marceau et al., 2016; Steinhart et al., 2017; Shifrut et al., 2018; Ihry et al., 2019).

More recently, newer iterations of CRISPR screens have emerged that utilized different forms of CRISPR-Cas9 system, which can transiently repress or activate gene expression instead of permanently editing the genome (Gilbert et al., 2014). In such systems, a nuclease-inactive form of Cas9 termed dead Cas9, or dCas9 is guided to specific sequences in the genome without introducing DSBs. In CRISPR interference (CRISPRi) screens, dCas9 is fused with a transcriptional repressor domain such as Krüppel associated box (KRAB) to suppress gene expression (Adamson et al., 2016; Liu et al., 2017; Ahmed et al., 2021). In contrast, CRISPR activation (CRISPRa) screens use dCas9 that is fused with a transcriptional activator domain such as VP64 to potentiate gene expression (Bester et al., 2018; Liu et al., 2018; Yang et al., 2019; Schmidt et al., 2022). CRISPRi and CRISPRa screens have a few advantages over the traditional CRISPR-KO screens. They are considered to be less toxic to cells because they do not introduce DNA breaks. While both CRISPRi and CRISPR-KO can cause loss of gene expression and function, CRISPRi has been proven to be more efficient than CRISPR-KO, as the latter generates a substantial fraction of inframe mutations (Mandegar et al., 2016). In addition, CRISPRa screens enable the interrogation of gene activation which may mimic gain-of-function mutations. On the other hand, CRISPRi and CRISPRa screens also have a few drawbacks and complications compared to CRISPR-KO screens. For example, both require the continuous expression of dCas9 protein to maintain suppression or activation. The use of strong transcriptional repressor or activator potentially can also introduce more long-lasting, unintended epigenetic modifications (Kampmann, 2018; Bock et al., 2022).

Several other emerging CRISPR-based genetic or epigenetic editing techniques are starting to be used for screens. CRISPR base editors, including cytosine base editors or adenosine base editors, allow precise genome editing at the nucleotide-level resolution without introducing DSBs. Collectively, they allow all four transition mutations (C to T, A to G, T to C, and G to A) by using a catalytically inactive Cas9 nickase that is fused to a base-modifying enzyme (Gaudelli et al., 2017; Rees and Liu, 2018). In an updated format, CRISPR prime editing enables precise DSB-independent genome modifications beyond the four base mutations. Prime editors employ a Cas9 nickase fused to an engineered reverse transcriptase and a prime-editing gRNA that serves not only to target the Cas9 protein to desired genomic regions, but also carries a template sequence with the desired sequence changes (Anzalone et al., 2019; Kantor et al., 2020). To date, a few reports have utilized base and prime CRISPR editing pooled screens to map DNA regulatory elements and to functionally assay disease-linked genetic variants (Cheng et al., 2021; Erwood et al., 2022). CRISPR technologies have also expanded beyond genome editing and transcriptional activation/repression into epigenome editing. CRISPR-based regulation of gene expression through precise epigenetic modifications is now feasible by generating a dCas9 protein that is fused to epigenetic effector proteins, such as DNMT3A and TET1, which regulate DNA methylation (Nakamura et al., 2021). CRISPRoff/on is one of the recent iterations of epigenetic CRISPR editing tools that establish DNA methylation to silence genes (CRISPRoff) or demethylation to reverse silencing (CRISPRon) (Nuñez et al., 2021). Genome-wide essentiality screens have been successfully conducted using the CRISPRoff system and were capable of silencing a large majority of protein-coding genes (Nuñez et al., 2021).

### Different CRISPR screening formats and readouts

A typical CRISPR-based functional genomics screen involves several key components; a tissue or a cell line, a sgRNA library to introduce the genetic perturbations, and an output assay. The Cas9 protein is either ectopically introduced with the sgRNA vector or stably expressed in the cells. The sgRNA library is usually delivered by lentiviral transduction and can be introduced in either an arrayed or a pooled format (Ford et al., 2019). In arrayed screens, each lentiviral sgRNA vector is prepared separately and transduced to cells in an arrayed format, typically in multi-well plates. Since the identity of the perturbation is known, different readouts are feasible to examine phenotypes of interest (Agrotis and Ketteler, 2015). Growing efforts are being made to generate genome-wide arrayed CRISPR libraries (Erard et al., 2017; Yin et al., 2022). However, arrayed screens have limited throughput in terms of readout analysis, and they are often costly and labor-intensive (Bock et al., 2022). On the other hand, pooled CRISPR screens enable genome-scale perturbations, because a pooled library of thousands to hundreds of thousands of sgRNA viral vectors are prepared, transduced, and assayed together. Therefore, a faster screening process can be achieved. Most pooled CRISPR screens to date involve competition among transduced cells under a biological challenge, which can be cellular survival under standard culture conditions, in response to a drug treatment or viral infection. The readout of such screens is the relative sgRNA abundances in the surviving population compared to the starting population as measured by next-generation sequencing. In a negative selection screen, the sgRNAs that dropped out from the remaining population are determined. Such screens have been frequently conducted to determine the fitness genes in different cancer cell lines (Hart et al., 2015; Tzelepis et al., 2016) as well as in hPSCs (Ihry et al., 2019; Mair et al., 2019). On the other hand, a positive selection screen aims to determine the sgRNAs that remain in the final population. A common application for such screens is to determine the genetic factors that confer resistance to therapeutic agents (Shalem et al., 2014) or viral infection (Marceau et al., 2016; Park et al., 2017; Li et al., 2019).

Depending on the aim of the study (positive or negative selection), the design of the screen usually varies. For example, traditional negative selection screens require a high library coverage (how many cells represent one gRNA) to detect genes that dropped out with high sensitivity, while less coverage is often needed in positive selection to determine enriched gRNAs. Recent versions of genome-wide CRISPR libraries have generated optimized sgRNA libraries with improved efficiencies and reduced off-target activities (Doench et al., 2016; Hart et al., 2017; Henkel et al., 2020). Combined with the improved algorithms for data analysis (Wang et al., 2019; Kim and Hart, 2021), it is now feasible to perform essentiality screens with higher dynamic range and reduced library coverage. It is worth mentioning that by adjusting the dynamic range of a CRISPR screen, it is possible to perform both positive and negative selections in a single screen. For example, a genome-wide CRISPR KO screen identified both positive and negative regulators of T-cell proliferation following stimulation (Shifrut et al., 2018).

In addition to assessing the impact of sgRNA on cell survival, cells used in a CRISPR screen can also be sorted and analyzed based on other phenotypes such as the expression profiles of cellular markers. In this type of screen, a protein of interest is either endogenously tagged with a fluorescent marker or detected with an antibody. Then, the perturbed cell populations are sorted into high- and low-expressing populations, which are later sequenced for sgRNA abundancies (Ihry et al., 2019; Bar et al., 2021; Condon et al., 2021).

Until recently, the read-outs from pooled CRISPR screens have been limited to variations in sgRNA distribution. However, the advances in single-cell transcriptomics and high-throughput imaging have allowed high-content readouts (Bock et al., 2022). Pooled CRISPR screens coupled with single-cell RNA sequencing, commonly referred to as single-cell CRISPR (scCRISPR) screens, such as CROP-seq/Perturb-seq/CRISPR-seq, allow multidimensional examination of signaling pathways that underlie different perturbations (Adamson et al., 2016; Dixit et al., 2016; Jaitin et al., 2016; Datlinger et al., 2017; Replogle et al., 2020). Depending on the design of the sgRNA vector or the technology used for single-cell RNA sequencing library preparation, sgRNA identities can be deconvoluted and assigned for single-cell transcriptomes. Pooled CRISPR screens with high-content imaging readouts have also become feasible. Following lentiviral library transduction, the desired cellular phenotypes are imaged either on fixed or live cells, and the perturbation identities of candidate cells are determined by different methods. Several studies utilized a photoactivable fluorescent protein, which is selectively activated in cells with the desired phenotype. These cells are later enriched with fluorescence-activated cell sorting (FACS) and sgRNA sequences are determined by sequencing (Kanfer et al., 2021; Yan et al., 2021). Another way to identify sgRNA sequences is by culturing cells on microraft arrays and isolating individual rafts with clones carrying the desired phenotypes for sgRNA sequencing (Wheeler et al., 2020). Alternatively, sgRNA sequences or associated barcodes are identified by *in situ* sequencing (Feldman et al., 2019; Funk et al., 2022).

## CRISPR screens in human PSC-derived neural cultures

Over the past decade, different formats of CRISPR-based screens have shed valuable insights into the molecular factors that underlie neurodevelopment and disease. Next, we will review recent literature that utilized CRISPR screens in hPSC-derived 2D and 3D neural cultures to study mechanisms of survival, cell fate specification, viral-host response, as well as neurodevelopmental and degenerative disorders (summarized in Table 1).

**TABLE 1:** Small and large-scale CRISPR screens that utilized hPSC-derived cultures to understand neurodevelopment and disease.

Technology	Target cells	Main library	Readout	Purpose	Reference
**Small scale**					
CRISPR-KO	hiPSCs differentiated into cerebral organoids	Pooled library targeting 20 human transcription factors	Single-cell RNA sequencing	Identification of neurodevelopmental fate decision regulators	Fleck et al. (2022)
CRISPR-KO	hESCs differentiated into cerebral organoids	Pooled library targeting 36 ASD genes	Single-cell RNA sequencing	Identification of developmental states and cell types susceptible to ASD perturbations	Li et al. (2022)
CRISPR-KO	hESCs differentiated into cerebral organoids	Pooled library targeting 172 microcephaly candidate genes	Quantification of sgRNA abundances	Identification of genes directly linked to microcephaly	Esk et al. (2020)
CRISPR-KO	hiPSC differentiated into subpallial and cortical organoids to generate forebrain assembloids	Pooled library targeting 425 genes linked to neurodevelopmental disorders including ASD	FACS and quantification of sgRNA abundances	Identification of regulators of human interneuron generation and migration.	Meng et al. (2022)
CRISPR-KO	hiPSCs-derived neural progenitors that were differentiated into cortical neurons	Pooled kinome-wide library targeting 736 kinases	Quantification of sgRNA abundances	Identifications of modifiers of neuronal survival in response to DPR-mediated toxicity	Guo et al. (2022)
**Large scale**					
CRISPRa	hiPSCs	Pooled library targeting 1,496 putative human transcription factors	FACS and quantification of sgRNA abundances	Identification of neuronal cell fate modulators	Black et al. (2020)
CRISPRi	hiPSCs differentiated into glutamatergic neurons	Pooled library targeting 2,325 genes representing the “druggable genome”	Quantification of sgRNA abundances for primary screens.	Identification of regulators of neuronal survival	Tian et al. (2019)
			Single-cell RNA sequencing and imaging in secondary validation screens		
CRISPRi/a	hiPSCs differentiated into microglia	Pooled library targeting 2,325 genes representing the “druggable genome”	Quantification of sgRNA abundances with or without FACS for primary screens.	Identification of regulators of microglia survival, activation, and synaptosome phagocytosis	Dräger et al. (2022)
			Single-cell RNA sequencing for CROP-seq validation screen.		
CRISPRi	hiPSC-derived astrocytes	Pooled library targeting 2,325 genes representing the “druggable genome”.	FACS and quantification of sgRNA abundances in primary screens.	Identification of regulators of inflammatory reactivity in astrocytes challenged with inflammatory cytokines	Leng et al. (2022)
		Pooled library targeting human transcription factors	Single-cell RNA sequencing for CROP-seq validation screen.		
CRISPR-KO	hPSC-derived neural progenitors	Pooled genome-wide library	Quantification of sgRNA abundances	Identification of factors that confer resistance against ZIKV infection	Li et al. (2019)
CRISPRi/a	hiPSCs differentiated into glutamatergic neurons	Pooled genome-wide library	Quantification of sgRNA abundances for primary screens.	Identification of regulators of neuronal survival in normal conditions and in response to oxidative stress	Tian et al. (2021)
			Single-cell RNA sequencing for a CROP-seq validation screen		

### Identifying drivers of neural cell fate specification

One of the powerful applications of CRISPR screens is to dissect regulators of cell fate specification. In two separate reports, large-scale CRISPRa screens were performed in human and mouse ESCs to activate the expression of all predicted transcription factors, either separately or in combination, with the aim of identifying neuronal cell fate modulators (Liu et al., 2018; Black et al., 2020). In both screens, a neuron-specific fluorescent reporter was used to enrich for neuronal and non-neuronal populations in the final population and determine transcription factors that promote or suppress neuronal differentiation, respectively. Both screens identified similar neurogenic factors including NEUROG3 and PRDM1, together with other candidate hits that were unique to each screen. Interestingly, different transcription factors can generate neurons with unique identities and signatures. For example, overexpression of Ezh2 generated neurons carrying floor plate markers (Liu et al., 2018), and ATOH1 activation generated neurons with dopaminergic activity (Black et al., 2020). Furthermore, combinatorial screens revealed how simultaneous activation of two transcription factors could enhance neuronal differentiation or promote synaptic maturation.

Single-cell CRISPR screen has also been successfully applied in 3D human iPSC-derived neural cultures to determine cell fate regulators during brain development. In this screen, a pooled library targeting 20 transcription factors was transduced into hPSCs and later differentiated into brain organoids carrying these perturbations in a mosaic fashion (Fleck et al., 2022). Single-cell transcriptomes of 2-months old organoids revealed the antagonistic roles of GLI3 and HES1 in shaping the dorsoventral telencephalon, where GLI3 activated cortical transition genes, while HES1 suppressed them. This study proposed an intriguing model where sonic hedgehog (SHH) signaling in neural progenitor cells induces GLI3, which in turn modulates HES genes to promote cortical fate acquisition. This coincides with another genome-wide CRISPR-KO screen which also revealed that SHH and ciliogenesis are among the key signaling pathways involved in the proper differentiation of neural progenitor cells from human ESCs by maintaining the organization of neural rosettes (Sivakumar et al., 2022).

### Understanding the fitness landscape of human neurons

Utilizing functional genomics to understand survival factors in human neurons can provide important insights into the mechanisms of selective vulnerability in neurodegenerative disorders. In 2019, a large-scale CRISPRi screen targeting the “druggable genome” was conducted in hPSCs, which were later differentiated into neurons to determine neuron-specific essential genes and compare them to hPSC-specific ones (Tian et al., 2019). This screen revealed factors that compromised neuronal survival upon knockdown, such as sterol metabolism-associated genes. In addition, genes that promoted neuronal survival upon knockdown were identified, such as those involved in the DLK/JNK pathways. Subsequent single-cell and imaging-based CRISPR screens that targeted the top hits revealed signaling pathways downstream of the candidate hits and showed additional cellular phenotypes, such as changes in neurite morphology and length. In a complementary study, a genome-wide CRISPRi/CRISPRa screen revealed the selective vulnerability of human neurons to oxidative stress due to the perturbations in genes that maintain redox balance (Tian et al., 2021). By utilizing oxidative stress as a challenge in a secondary screen, Tian R. et al. showed that the knockdown of one of the strongest hits, the lysosomal protein prosaposin (PSAP), compromised neuronal survival through lysosomal failure that triggerred ferroptosis in neurons. Collectively, these studies were the first to perform a large-scale CRISPR-based screen in human hPSC-derived neurons. They generated rich lists of genetic modulators of neuronal survival and single-cell transcriptomic datasets that can be examined in future studies to unravel new therapeutic targets for neurodegenerative diseases.

### Examining the regulation of immune responses in glial cells

Recent studies on hPSC-derived glial cells have harnessed the power of CRISPR screens to uncover the regulators of survival and inflammatory response. Astrocytes and microglia are non-neuronal brain cells that play essential roles in brain development and homeostasis, and they are considered the main components of the neuroinflammatory response (Salter and Stevens, 2017; Vasile et al., 2017; Garland et al., 2022). Both cell types can adopt different reactive states in response to brain injury or diseases, which can have neuroprotective or neurotoxic consequences (Liddelow et al., 2017; Franklin et al., 2021). CRISPRi/a screens in hPSC-derived microglia proposed microglia-specific survival factors such as members of the colony stimulating factor (CSF) receptor family (Dräger et al., 2022). By sorting the perturbed populations using markers of inflammatory response or phagocytosis, this study also uncovered modifiers of microglia activation including CDK12 and MED1, and modifiers of synaptosome phagocytosis such as CD209. Additionally, a CROP-seq screen showed regulators of different microglia transcriptional states, including a state characterized by chemokine signatures specific to the human brain (Geirsdottir et al., 2019). Most importantly, the screen by Drager et al. identified a microglia state characterized by Osteopontin (SPP1) expression, a marker known to be upregulated in disease-activated microglia such as Alzheimer’s (Sala Frigerio et al., 2019) and multiple sclerosis (van der Poel et al., 2019). The SPP1+ microglia state was found to be selectively vulnerable to CSF1R knockdown, a key finding that paves the road for future therapeutic interventions that can manipulate such disease-associated microglia.

A similar screen was utilized to identify immune response modifiers in hPSC-derived astrocytes (Leng et al., 2022). Neurotoxic reactive cytokines are often induced in response to pro-inflammatory cytokines released after CNS injury and disease (Guttenplan et al., 2020a; Guttenplan et al., 2020b). By challenging human astrocytes with the inflammatory cytokines IL-1α/TNF/C1q, pooled CRISPRi screens revealed regulators of immune response such as the canonical NF-kB, interferon, and acute phase response signaling. A complementary CROP-seq screen revealed that NF-kB signaling activates two different inflammatory reactive astrocyte signatures (IRAS) marked by distinct responsive genes that act in an autocrine-paracrine fashion. IRAS1 is activated by STAT3 and marked by IL-1/IL-6 signaling genes, while IRAS2 is inhibited by STAT3 and marked by TNF/IFN-responsive genes. Taken together, CRISPR screens in human glial cultures have provided valuable insights into the important regulators of immune response, which will set the stage for future research that dissects disease-associated glial reactivity in neurological diseases.

### Revealing the modifiers of host-virus interactions

Neurovirulent viruses such as Zika (ZIKV), Dengue (DENV), and Japanese encephalitis (JEN) can infect different brain cells including neurons, neural progenitors, and glial cells, leading to severe neurological manifestations and brain malformations in newborn children (Chen et al., 2019). CRISPR screens have identified several key host factors that are required for virus infections, thereby providing promising targets for antiviral treatment (Labeau et al., 2020; Chulanov et al., 2021; Hoffmann et al., 2021). However, most published reports were performed on cancer cell lines. Therefore, host resistance factors in specific brain-relevant cell types might not have been identified. Recent studies have reported genome-wide CRISPR-KO screens that identified ZIKV host factor genes in hPSC-derived neural progenitor cultures (Li et al., 2019; Wells et al., 2023). In these screens, mutations in genes involved in heparan sulfation, endocytosis, ER processing, and Golgi function conferred resistance against ZIKV in neural progenitors. These studies identified known suppressors of Interferon (IFN) signaling such as ISG15 and SOCS3, a finding echoed by a separate CRISPRa screen that showed interferon-stimulating genes such as IFN-λ2 and ISI6 among the top hits that, when upregulated, protected cancer cells from ZIKV (Dukhovny et al., 2019). Interestingly, CRISPR-KO screens in human neural progenitors identified ZIKV-dependent factors that were not previously reported in cancer cell screens, such as the vacuolar ATPase subunits (V-ATPase) involved in endosome-lysosome acidification (Li et al., 2019; Wells et al., 2023). Such findings emphasize the importance of performing CRISPR screens to investigate viral-host interactions in brain-relevant cell types. Indeed, to identify factors involved in ZIKV neurotropism, Wang S. et al. Conducted a genome-wide CRISPR-KO screen in glioblastoma stem cells to represent neural stem cell-like cells and compared the hits to those from a parallel screen in HEK293FT (Wang et al., 2020) to identify neuro-specific host factors. Surprisingly, the knockout of 92 candidate hits protected glioblastoma stem cells but not HEK293 cells from ZIKV infection, with ITGB5 among the top hits, which the authors then showed to mediate ZIKV internalization in human neural stem cells.

### Studying the genetic factors underlying neurodevelopmental and degenerative disorders

Genetic association studies have uncovered thousands of gene mutations linked to neurodevelopmental and neurodegenerative disorders. Understanding the molecular and cellular functions of these genes in a disease relevant context is essential to untangle the correlation between the disease genotypes and patient phenotypes. In addition, it will pave the road for developing diagnostic markers and therapeutic targets. Animal models of disease-linked mutations have shed valuable mechanistic insights, but such models may not fully translate to the human genetic landscape, and recapitulate the complex nature of the human brain. In this section, we review recent CRISPR functional screens performed in hPSC-derived 2D neural cultures and 3D brain organoids to untangle the biology of autism, microcephaly, and ALS.

#### Autism

Autism spectrum disorder (ASD) represents a set of neurodevelopmental disorders that are linked to over a thousand risk genes (Abrahams et al., 2013; Lai et al., 2014). This complex polygenic nature highlights the need for systematic functional genomics approaches to dissect how these mutations contribute to ASD pathology. In 2022, two separate CRISPR screens in hPSC-derived 3D brain organoid models have identified several convergent mechanisms and cellular abnormalities across different ASD-risk genes. A single-cell CRISPR screen in human telencephalon organoids examined the pooled knockout effect of 36 high-risk ASD genes involved in transcriptional control (Li et al., 2022). To establish this approach, the authors transduced human ESCs carrying an inducible Cas9 cassette with a pooled sgRNA lentiviral library. After embryoid body formation, gene knockout was induced, followed by neural induction to generate CRISPR-edited mosaic brain organoids. Single-cell RNA sequencing on 4-months old organoids revealed that many ASD perturbations disrupted dorsal-ventral cell populations. In the dorsal populations, intermediate progenitors were depleted, while in the ventral population, ventral radial glia cells (RGCs), interneuron progenitors, and lateral ganglionic eminence-derived interneurons were enriched. Such data implicated neural progenitor cells to be among the most susceptible cells in ASD pathology. This finding is consistent with an independent study in the *Xenopus*, which observed an increase in the ratio of neural progenitors to differentiating neurons in the telencephalon following CRISPR knockout of 10 high-risk ASD genes (Willsey et al., 2021).

Further analyses of the different cell states and developmental stages in the ASD organoid screens showed that ASD gene perturbations accelerated upper-layer neurogenesis and impaired ventral progenitor differentiation. In addition, the authors showed that perturbation in the BAF complex member ARID1B was among the strongest hits that expanded ventricular radial glia cells in the ventral telencephalon and biased their transition into oligodendrocyte precursor cells over neuronal cell fates. Interestingly, the findings of this screen aligned well with an *in vivo* perturb-seq study that examined the single-cell transcriptomic changes upon pooled CRISPR perturbation of 35 *de novo* ASD risk genes introduced in the developing mouse brain (Jin et al., 2020). This *in vivo* screen revealed that perturbations in ASD genes altered cell states in different brain cells with an upregulation of an interneuron module and accelerated maturation of oligodendrocytes. Such findings suggest that mutations in ASD genes impact different cell states in a manner that might be conserved in the mammalian brain. The involvement of ASD genes in neuronal differentiation has also been proposed by another CRISPRi screen that targeted 13 ASD genes in immortalized human neural progenitor cells, which were later differentiated into neurons (Lalli et al., 2020). Single-cell transcriptomic profiling showed that ASD genes converge onto two functionally distinct gene modules that either delay or accelerate neuronal differentiation.

A CRISPR-assembloid platform has been recently established to examine the role of ASD genes in interneuron migration and development (Meng et al., 2022). Abnormalities in interneuron development and function have been linked to ASD neuropathology (Contractor et al., 2021). However, it remains unclear which ASD genes regulate interneuron development. Towards this aim, Meng X. et al. Conducted a CRISPR-KO screen that targeted 425 genes linked to neurodevelopmental disorders including ASD in an hPSC line carrying a fluorescent reporter that labels ventral forebrain interneuron lineages. To determine which genes affected interneuron development, hPSCs were differentiated into subpallial organoids. Quantification of sgRNA abundances in the sorted cell populations identified SMAD4 and CSDE1 among 13 other hits that were required for interneuron patterning. To identify ASD genes involved in interneuron migration, a parallel elegant screen was performed in human forebrain assembloids generated by integrating unperturbed cortical organoids with the perturbed fluorescently-labeled subpallial ones. By comparing the enriched sgRNAs in the interneuron populations that migrated into the cortical organoids versus the ones that remained in the subpallial organoids, the authors identified LNPK, an ER-associated gene (Breuss et al., 2018), among the top hits involved in interneuron migration.

### Microcephaly

Microcephaly is a neurodevelopmental disorder characterized by reduced brain size, which can result from different genetic and environmental factors (Jayaraman et al., 2018). These different types of microcephaly have recently been successfully modeled in hPSC-derived brain organoids (Lancaster et al., 2013; Zhang et al., 2019; Dhaliwal et al., 2021), making the latter a promising culture system to conduct screens for additional microcephaly-related genes. However, the inherently unequal growth dynamics of different cell lineages in the brain organoids can complicate the quantification of sgRNA abundances as a readout for perturbation-mediated cell number changes. To overcome these limitations, Esk C. et al. Developed CRISPR- LIneage tracing at Cellular resolution in Heterogenous Tissue (CRISPR-LICHT) (Esk et al., 2020), which utilizes a dual barcoding approach to link sgRNA information to cell lineage numbers. Using this technique, human ESCs were transduced with a sgRNA library that targeted 172 microcephaly candidate genes and were then differentiated into brain organoids. The screen revealed 25 candidate genes that resulted in sgRNA depletion within the organoids. Many hits were involved in pathways commonly implicated in microcephaly such as centriole biogenesis and the DNA damage response. This study also characterized a mutation in the Immediate Early Response 3 Interacting Protein 1 (IER3IP1) gene that have not been previously linked to microcephaly. Functionally, this gene is involved in the unfolded protein response (UPR) and extracellular matrix (ECM) deposition. The dysregulation of these pathways likely leads to disrupted localization of neural progenitors and premature neurogenesis, which are some of the common cellular pathologies in microcephaly (Zaqout and Kaindl, 2022). The CRISPR-LICHT screen not only offers new microcephaly linked genes for future loss of function studies, but also provides a scalable CRISPR screening platform that utilizes organoids approaches to identify brain size determinants.

### C9orf72-associated neurodegeneration

A hexanucleotide repeat (G_4_C_2_) expansion in the *C9ORF72* gene represents one of the most common causes of frontotemporal dementia (FTD) and Amyotrophic lateral sclerosis (ALS) (DeJesus-Hernandez et al., 2011; Renton et al., 2011; Balendra and Isaacs, 2018). This expansion usually results in aggregated dipeptide repeats (DPRs) that represents one of the hallmarks of FTD-ALS neuropathology (Freibaum and Taylor, 2017). Previous CRISPR screens have utilized diverse approaches in human cancer or transformed cell lines to identify modifiers of different processes involved in G_4_C_2_ repeat toxicity (Kramer et al., 2018; Cheng et al., 2019; Chai et al., 2020). By utilizing exogeneous or endogenous DPRs as a challenge, Kramer NJ. et al. Showed that mutations in the endosomal trafficking gene RAB7 and the ER-associated gene TMX2 protected human cells and primary neurons from PR toxicity (Kramer et al., 2018). Another CRISPR screen utilized a DPR fluorescent reporter and FACS-based approaches to identify modulators of DPR production. This study identified the RNA helicase DDX3X among the top hits that, upon knockout, increased endogenous DPR levels (Cheng et al., 2019). Another screen transduced a C9orf72 knockout human cell line with a genome-wide CRISPR library to identify factors that genetically interact with C9orf72. Quantification of sgRNA abundance in the remaining populations identified the mitochondrial membrane protein FIS1 among the strongest synthetic lethal genetic interactors (Chai et al., 2020). These studies provided a wealth of information regarding C9orf72-mediated pathology. However, because the primary screens were conducted in non-neuronal cells, they could have missed some genetic modifiers that only function in disease-relevant neural cell types.

In 2022, Guo W. et al. conducted the first kinome-wide CRISPR screen in hPSC-derived cortical neurons to identify DPR toxicity modifiers. They found that knockouts in 113 genes, which are known to be involved in axon regeneration, dendrite development, and cytoskeletal organization, enhanced neuronal survival under DPR treatment (Guo et al., 2022). Furthermore, this study revealed that inhibiting NEK6, one of the top hits, prevented DPR-induced neuronal cell death and rescued axonal transport defects by inhibiting P53-mediated DNA damage. Future studies that utilize genome-wide CRISPR screens in hPSC-derived neuronal cells from FTD-ALS can help (Fujimori et al., 2018; Aldana et al., 2020) provide novel insights into the genetic modifiers that can be targeted for therapeutic purposes.

## Experimental considerations, limitations, and future directions

**FIGURE 2:**
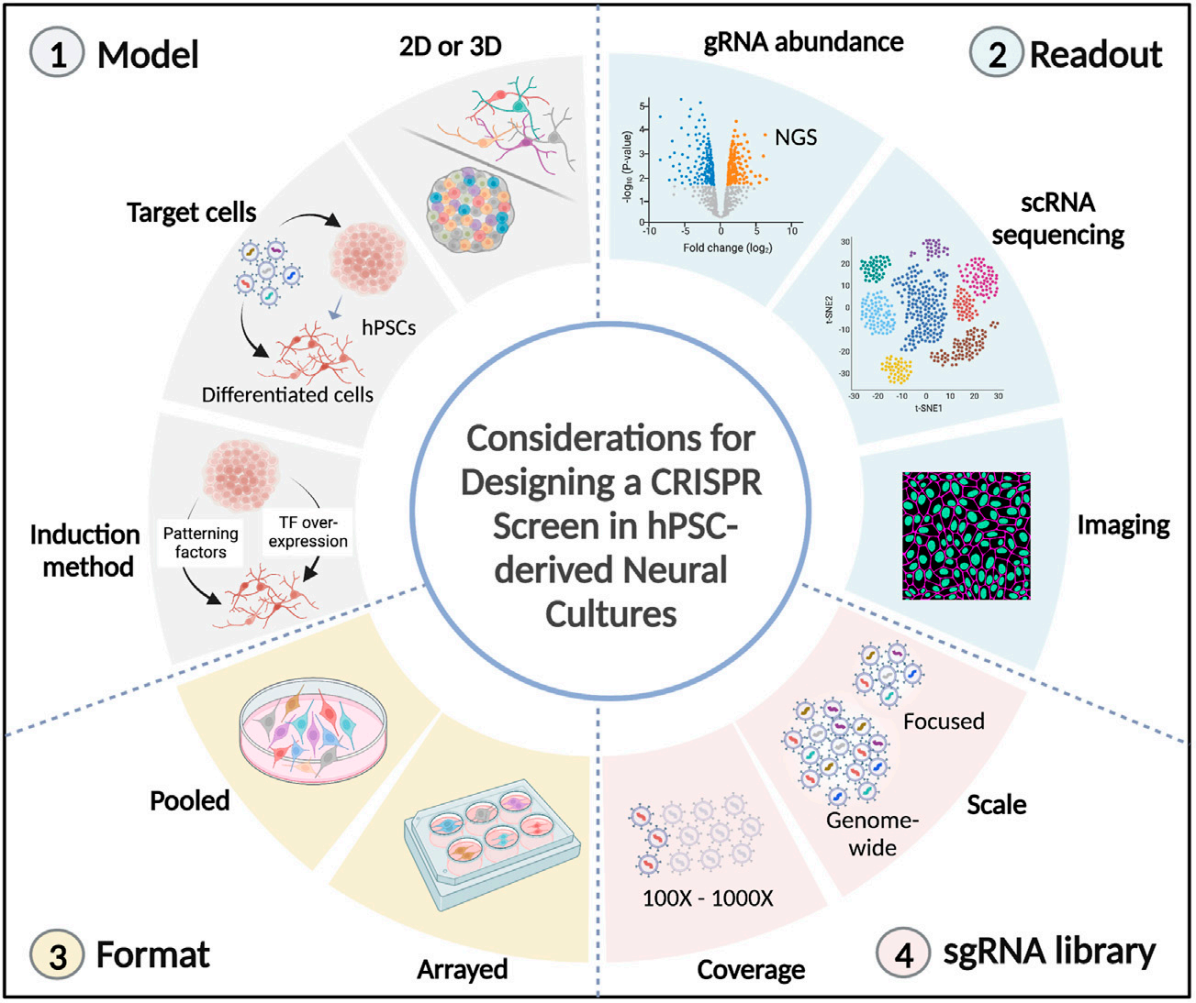
Technical considerations in CRISPR screens using hPSC-derived neural cultures. Several factors need to be considered in the design of a CRISPR screen using hPSC-derived neural cultures. 1) The model to be investigated. It can either be 2D (neural progenitors, neurons, or glial cells) or 3D (organoids/assembloids), the stage during which perturbations are induced (hPSCs or differentiated cells), and the differentiation method needs to be considered (extrinsic patterning factors or overexpression of transcription factors). 2) The desired readout. It can be the quantification of sgRNA abundances (following competition for cell survival or cell sorting), or high-content readouts, such as single-cell transcriptomics and spatial imaging. 3) The format of the screen. Pooled perturbations can be introduced to target cells in bulk or in an arrayed format (1 perturbation/well). 4) The sgRNA library. The scale of genes to be targeted (it can be genome-wide or a more focused library, such as the druggable genome, transcription factors or disease-risk genes). The gRNA coverage needs to be considered, which can range from 100 to 1000 fold representation for each sgRNA. TF: transcription factor, NGS: next-generation sequencing.

There are several factors to consider when designing a CRISPR screen using hPSC-derived neural cultures (Figure 2).

### Library scale, coverage, and choice of cells to be screened

Library scale is one of the first factors to consider when designing a CRISPR screen, and it largely depends on the aim of the screen. Genome-wide screens are comprehensive because they target the majority of protein-coding genes and they can uncover novel genes involved in basic biological processes or disease development. In addition, several genome-wide CRISPR libraries are readily-available (Hart et al., 2015; Doench et al., 2016; Henkel et al., 2020), reducing the steps needed to design and clone the gRNA library (Otten and Sun, 2020). However, genome-wide screens can be costly and labor-intensive, in particular, when applied in complex models (e.g., brain organoids) or with high-content readouts (such as scRNA sequencing). Alternatively, more focused libraries can be utilized to target the druggable genome (Tian et al., 2019), transcription factors (Black et al., 2020), or disease-linked genes (Esk et al., 2020; Li et al., 2022; Meng et al., 2022).

Library coverage reflects how often each sgRNA is represented in the targeted cellular population. Most screens utilize a library coverage between 100 and 1000 folds. Reaching this coverage for genome-wide screens usually means that hundreds of millions of cells are needed for the library transduction. Depending on the specific neural cell types, achieving this number of cells could be challenging as some hPSC-derived neural cultures may have a more limited expansion potential post-differentiation. Additionally, some neural cells such as microglia can be more difficult to transduce. To overcome these restrictions, introducing the library in hPSCs as opposed to the target cell type could be a feasible alternative as hPSCs can be readily transduced and expanded. However, one needs to be mindful that generating perturbations in the hPSC stage could potentially complicate the interpretation of data as candidate hits might arise due to defects in hPSC differentiation. The use of hPSC lines that express an inducible Cas9 gene has helped overcome such challenges by controlling Cas9 expression during or after differentiation (Tian et al., 2019; Esk et al., 2020; Dräger et al., 2022; Li et al., 2022). Additional challenges associated with hPSCs need to be taken in consideration, such as possible transgene silencing in the pluripotent and/or differentiated cell states (Ordovás et al., 2015; Cabrera et al., 2022), and hPSC population bottlenecking that might arise due to significant cell loss from Cas9-induced double-strand breaks (Ihry et al., 2018).

Another factor to consider in the design of a CRISPR screen is the method utilized to differentiate hPSCs into neuronal or glial cells. Although some of the above-mentioned CRISPR screens utilized classic differentiation protocols based on introducing extrinsic signals (Shi et al., 2012; Guo et al., 2022), most reports favor approaches that overexpress different cell-fate determining transcription factors such as NGN2 to generate neurons (Zhang et al., 2013; Tian et al., 2019), or NFIA and SOX9 to generate astrocytes (Li et al., 2018; Leng et al., 2022). The latter approach is relatively fast, and often occurs within days, making it more convenient for screening. However, there are several points to consider when utilizing transcription-factor induction approaches (Flitsch et al., 2020; Hulme et al., 2021). First, it is important to validate how much the differentiated cells physiologically resemble those obtained from extrinsic factor-based models. In addition, the cells generated from transcription factor overexpression might not represent a pure population. For example, Lin et al. showed that heterogenous populations of neurons were generated from NGN2 overexpression in different iPSC lines (Lin et al., 2021). However, this limitation can be overcome by combining NGN2 overexpression with different patterning factors (Chen et al., 2020).

### 2D, 3D, or *in vivo* screens?

To date, most CRISPR screens in hPSC-derived neural cultures utilized 2D monoculture models because they are scalable, less liable to variations, and have relatively homogeneous growth dynamics. On the other hand, hPSC-derived 2D cultures do not recapitulate the intricate organization and heterogeneity of the brain. Therefore, are not suitable for studying complex phenotypes, which include cell-cell interactions and cell migration. Such phenotypes can be examined using screens in hPSC-derived organoids because they better mimic the different aspects of early human brain development. Furthermore, organoids have been used successfully to model many disorders which include ASD, Rett syndrome, microcephaly, Alzheimer’s, and Parkinson’s, among other diseases (Tian et al., 2020; Eichmüller and Knoblich, 2022), thereby providing an appropriate disease context if needed. One drawback of the earlier iterations of protocols for generating brain organoids is that they may not contain complex neuronal circuitry and often lack vascular and immune cells. Such challenges can be overcome by generating assembloids that exhibit functional neuronal circuitry between organoids of different brain regions (Miura et al., 2020), co-culturing organoids with microglia that can affect brain organoid development under physiological and pathological conditions (Muffat et al., 2018; Popova et al., 2021), or improving organoid vasculature by co-culturing them with endothelial cells (Pham et al., 2018).

While hPSC-derived 2D and 3D neural systems are well suited to recapitulate features of the developing fetal brain, it could be challenging to use them to model the aging brain and its related disorders. In this regard *in vivo* CRISPR screens are gaining traction (Wertz et al., 2020; Ruetz et al., 2021). A recent study utilized both *in vitro* and *in vivo* CRISPR screening platforms in old mice and uncovered over 300 factors that boosted the activation of aged neural stem cells (NSCs) (Ruetz et al., 2021). The *in vivo* screen was useful to validate top hits from the *in vitro* screen performed in primary NSCs from old mice. It is worth mentioning that the library size in *in vivo* screens is usually limited to a few hundred genes because delivering CRISPR library components effectively into sufficient quantities of the cells of interest can be challenging (Kuhn et al., 2021).

### Format (arrayed vs. pooled) and their readouts

The choice between arrayed and pooled CRISPR-based screens in hPSC-derived cultures depends on the phenotype to be examined and the desired readout. Different readouts can be assayed in arrayed screens. In addition, minimal deconvolution is needed to link genotypes with the obtained phenotypes. However, due to challenges of analyzing readouts in arrayed formats, arrayed screens are usually limited in the number of perturbations that can be examined. Therefore, secondary CRISPR screens with arrayed formats are commonly performed in conjunction with pooled large-scale screens to validate their top hits. Pooled CRISPR screens coupled with examining sgRNA variations in surviving populations remain the gold standard to probe basic brain development and the mechanisms underlying neuropathology. As we discussed, several reports have successfully used this technology in hPSC-derived neurons, glial cells and even organoids and assembloids to identify regulators of neuronal cell fate specifications, cell survival, and disease phenotypes associated with ASD and microcephaly. Previous functional genomics screens in immortalized human cell lines provided valuable mechanistic insights into the modulators of different pathologies associated with neurodegenerative diseases such as Alzheimer’s and Parkinson’s disease (Potting et al., 2018; Rousseaux et al., 2018; Sanchez et al., 2021). Future work can extend the findings of those studies by utilizing hPSC-derived disease-relevant cell types. For instance, CRISPR screens on hPSC-derived dopaminergic neurons carrying Parkinson’s Disease-associated SNCA mutations (Michel et al., 2016) or hPSC-derived motor neurons harboring mutant forms of TARDBP linked to familial ALS (Bilican et al., 2012) can provide novel genetic modifiers that promote survival of these selectively-vulnerable neuronal cell types.

Utilizing pooled screens in 3D brain organoids with readouts that depend on sgRNA enrichment/depletion has been challenging, probably owing to the large library coverage needed to overcome noise associated with the heterogenous growth dynamics in 3D cultures. Luckily, recent CRISPR screens in brain, colon, and intestinal organoids have helped overcome these challenges by employing clonal tracing and single organoid sequencing technologies (Esk et al., 2020; Michels et al., 2020; Ringel et al., 2020). The development of these new CRISPR screening technologies will aid in probing phenotypes that can be uniquely modeled in human brain organoids, such as examining regulators of human brain size expansion and folding (Li et al., 2017), and genetic modifiers of brain size reduction upon challenging organoids with insults such as ZIKV infection (Garcez et al., 2016).

The introduction of CRISPR screens with single-cell transcriptomic readouts have unleashed unprecedented research avenues that can utilize 2D and 3D hPSC-dervied neural cultures. By integrating findings from genome-wide association studies, such screens can provide valuable insights into the complex biology of polygenic neuropsychiatric disorders, which is difficult to discern with single-mutant studies. For example, It is estimated that over a hundred loci and more than a thousand risk genes are linked to schizophrenia (Allen et al., 2008; Harrison, 2015). By employing single-cell CRISPR screens that target the high-risk genes in brain organoids (Notaras et al., 2022), it is possible to identify common molecular pathways that might be involved in the pathology of schizophrenia. Furthermore, one can delineate the different cell types and states affected by the different perturbations. Imaging-based CRISPR screens are ideally suited to resolve many of the phenotypes examined in neural cultures, such as neuronal and glial morphologies, synaptogenesis, and protein localization/aggregation. It is expected that this technology will be employed in future studies to identify genetic modifiers of different proteinopathies associated with neurodegeneration such as tau, TDP43, *ß*-amyloid, and *a*-synuclein among others (Marsh, 2019).

In conclusion, the combination of CRISPR-based screens and hPSC-derived neural systems has revolutionized our knowledge of the complex interactions between genetics, development, and disease. It is expected that the persistent advancements in CRISPR technologies, high-content readouts, and methods to model human brain development *in vitro* will continue to drive significant progress in our understanding of basic brain biology and the genetic underpinnings of neurological disorders. In the future, the findings obtained from CRISPR screens in hPSC-derived cultures are anticipated to extend beyond basic science and will be utilized in translational research to target genetic modifiers of disease-linked proteins or reactive cell states, which can improve treatment outcomes for various neurological diseases.

## References

[B1] Abrahams B. S. Arking D. E. Campbell D. B. Mefford H. C. Morrow E. M. Weiss L. A. (2013). SFARI gene 2.0: A community-driven knowledgebase for the autism spectrum disorders (ASDs). Mol. Autism 4, 36. 10.1186/2040-2392-4-36 24090431 PMC3851189

[B2] Abud E. M. Ramirez R. N. Martinez E. S. Healy L. M. Nguyen C. H. H. Newman S. A. (2017). iPSC-derived human microglia-like cells to study neurological diseases. Neuron 94, 278–293. e9. 10.1016/j.neuron.2017.03.042 28426964 PMC5482419

[B3] Adamson B. Norman T. M. Jost M. Cho M. Y. Nuñez J. K. Chen Y. (2016). A multiplexed single-cell CRISPR screening platform enables systematic dissection of the unfolded protein response. Cell. 167, 1867–1882. e21. 10.1016/j.cell.2016.11.048 27984733 PMC5315571

[B4] Agrotis A. Ketteler R. (2015). A new age in functional genomics using CRISPR/Cas9 in arrayed library screening. Front. Genet. 6, 300. 10.3389/fgene.2015.00300 26442115 PMC4585242

[B5] Ahmed M. Soares F. Xia J.-H. Yang Y. Li J. Guo H. (2021). CRISPRi screens reveal a DNA methylation-mediated 3D genome dependent causal mechanism in prostate cancer. Nat. Commun. 12, 1781. 10.1038/s41467-021-21867-0 33741908 PMC7979745

[B6] Aldana B. I. Zhang Y. Jensen P. Chandrasekaran A. Christensen S. K. Nielsen T. T. (2020). Glutamate-glutamine homeostasis is perturbed in neurons and astrocytes derived from patient iPSC models of frontotemporal dementia. Mol. Brain 13, 125. 10.1186/s13041-020-00658-6 32928252 PMC7491073

[B7] Allen N. C. Bagade S. McQueen M. B. Ioannidis J. P. A. Kavvoura F. K. Khoury M. J. (2008). Systematic meta-analyses and field synopsis of genetic association studies in schizophrenia: The SzGene database. Nat. Genet. 40, 827–834. 10.1038/ng.171 18583979

[B8] Anzalone A. V. Randolph P. B. Davis J. R. Sousa A. A. Koblan L. W. Levy J. M. (2019). Search-and-replace genome editing without double-strand breaks or donor DNA. Nature 576, 149–157. 10.1038/s41586-019-1711-4 31634902 PMC6907074

[B9] Austin C. P. Battey J. F. Bradley A. Bucan M. Capecchi M. Collins F. S. (2004). The knockout mouse project. Nat. Genet. 36, 921–924. 10.1038/ng0904-921 15340423 PMC2716027

[B10] Bagley J. A. Reumann D. Bian S. Lévi-Strauss J. Knoblich J. A. (2017). Fused cerebral organoids model interactions between brain regions. Nat. Methods 14, 743–751. 10.1038/nmeth.4304 28504681 PMC5540177

[B11] Balendra R. Isaacs A. M. (2018). C9orf72-mediated ALS and FTD: Multiple pathways to disease. Nat. Rev. Neurol. 14, 544–558. 10.1038/s41582-018-0047-2 30120348 PMC6417666

[B12] Bar S. Vershkov D. Keshet G. Lezmi E. Meller N. Yilmaz A. (2021). Identifying regulators of parental imprinting by CRISPR/Cas9 screening in haploid human embryonic stem cells. Nat. Commun. 12, 6718. 10.1038/s41467-021-26949-7 34795250 PMC8602306

[B13] Berns K. Hijmans E. M. Mullenders J. Brummelkamp T. R. Velds A. Heimerikx M. (2004). A large-scale RNAi screen in human cells identifies new components of the p53 pathway. Nature 428, 431–437. 10.1038/nature02371 15042092

[B14] Bester A. C. Lee J. D. Chavez A. Lee Y.-R. Nachmani D. Vora S. (2018). An integrated genome-wide CRISPRa approach to functionalize lncRNAs in drug resistance. Cell. 173, 649–664. e20. 10.1016/j.cell.2018.03.052 29677511 PMC6061940

[B15] Bilican B. Serio A. Barmada S. J. Nishimura A. L. Sullivan G. J. Carrasco M. (2012). Mutant induced pluripotent stem cell lines recapitulate aspects of TDP-43 proteinopathies and reveal cell-specific vulnerability. Proc. Natl. Acad. Sci. 109, 5803–5808. 10.1073/pnas.1202922109 22451909 PMC3326463

[B16] Black J. B. McCutcheon S. R. Dube S. Barrera A. Klann T. S. Rice G. A. (2020). Master regulators and cofactors of human neuronal cell fate specification identified by CRISPR gene activation screens. Cell. Rep. 33, 108460. 10.1016/j.celrep.2020.108460 33264623 PMC7730023

[B17] Bock C. Datlinger P. Chardon F. Coelho M. A. Dong M. B. Lawson K. A. (2022). High-content CRISPR screening. Nat. Rev. Methods Primer 2, 8. 10.1038/s43586-021-00093-4 PMC10200264 37214176

[B18] Boutros M. Ahringer J. (2008). The art and design of genetic screens: RNA interference. Nat. Rev. Genet. 9, 554–566. 10.1038/nrg2364 18521077

[B19] Breuss M. W. Nguyen A. Song Q. Nguyen T. Stanley V. James K. N. (2018). Mutations in LNPK, encoding the endoplasmic reticulum junction stabilizer lunapark, cause a recessive neurodevelopmental syndrome. Am. J. Hum. Genet. 103, 296–304. 10.1016/j.ajhg.2018.06.011 30032983 PMC6080764

[B20] Brockerhoff S. E. Hurley J. B. Janssen-Bienhold U. Neuhauss S. C. Driever W. Dowling J. E. (1995). A behavioral screen for isolating zebrafish mutants with visual system defects. Proc. Natl. Acad. Sci. U. S. A. 92, 10545–10549. 10.1073/pnas.92.23.10545 7479837 PMC40648

[B21] Cabrera A. Edelstein H. I. Glykofrydis F. Love K. S. Palacios S. Tycko J. (2022). The sound of silence: Transgene silencing in mammalian cell engineering. Cell. Syst. 13, 950–973. 10.1016/j.cels.2022.11.005 36549273 PMC9880859

[B22] Cao S.-Y. Hu Y. Chen C. Yuan F. Xu M. Li Q. (2017). Enhanced derivation of human pluripotent stem cell-derived cortical glutamatergic neurons by a small molecule. Sci. Rep. 7, 3282. 10.1038/s41598-017-03519-w 28607372 PMC5468244

[B23] Chai N. Haney M. S. Couthouis J. Morgens D. W. Benjamin A. Wu K. (2020). Genome-wide synthetic lethal CRISPR screen identifies FIS1 as a genetic interactor of ALS-linked C9ORF72. Brain Res. 1728, 146601. 10.1016/j.brainres.2019.146601 31843624 PMC7539795

[B24] Chen M. Maimaitili M. Habekost M. Gill K. P. Mermet-Joret N. Nabavi S. (2020). Rapid generation of regionally specified CNS neurons by sequential patterning and conversion of human induced pluripotent stem cells. Stem Cell. Res. 48, 101945. 10.1016/j.scr.2020.101945 32791483

[B25] Chen T. He X. Zhang P. Yuan Y. Lang X. Yu J. (2019). Research advancements in the neurological presentation of flaviviruses. Rev. Med. Virol. 29, e2021. 10.1002/rmv.2021 30548722 PMC6590462

[B26] Cheng L. Li Y. Qi Q. Xu P. Feng R. Palmer L. (2021). Single-nucleotide-level mapping of DNA regulatory elements that control fetal hemoglobin expression. Nat. Genet. 53, 869–880. 10.1038/s41588-021-00861-8 33958780 PMC8628368

[B27] Cheng W. Wang S. Zhang Z. Morgens D. W. Hayes L. R. Lee S. (2019). CRISPR-Cas9 screens identify the RNA helicase DDX3X as a repressor of C9ORF72 (GGGGCC)n repeat-associated non-AUG translation. Neuron 104, 885–898. e8. 10.1016/j.neuron.2019.09.003 31587919 PMC6895427

[B28] Chulanov V. Kostyusheva A. Brezgin S. Ponomareva N. Gegechkori V. Volchkova E. (2021). CRISPR screening: Molecular tools for studying virus–host interactions. Viruses 13, 2258. 10.3390/v13112258 34835064 PMC8618713

[B29] Condon K. J. Orozco J. M. Adelmann C. H. Spinelli J. B. van der Helm P. W. Roberts J. M. (2021). Genome-wide CRISPR screens reveal multitiered mechanisms through which mTORC1 senses mitochondrial dysfunction. Proc. Natl. Acad. Sci. U. S. A. 118, e2022120118. 10.1073/pnas.2022120118 33483422 PMC7848693

[B30] Contractor A. Ethell I. M. Portera-Cailliau C. (2021). Cortical interneurons in autism. Nat. Neurosci. 24, 1648–1659. 10.1038/s41593-021-00967-6 34848882 PMC9798607

[B31] Datlinger P. Rendeiro A. F. Schmidl C. Krausgruber T. Traxler P. Klughammer J. (2017). Pooled CRISPR screening with single-cell transcriptome readout. Nat. Methods 14, 297–301. 10.1038/nmeth.4177 28099430 PMC5334791

[B32] DeJesus-Hernandez M. Mackenzie I. R. Boeve B. F. Boxer A. L. Baker M. Rutherford N. J. (2011). Expanded GGGGCC hexanucleotide repeat in noncoding region of C9ORF72 causes chromosome 9p-linked FTD and ALS. Neuron 72, 245–256. 10.1016/j.neuron.2011.09.011 21944778 PMC3202986

[B33] Dhaliwal N. Choi W. W. Y. Muffat J. Li Y. (2021). Modeling PTEN overexpression-induced microcephaly in human brain organoids. Mol. Brain 14, 131. 10.1186/s13041-021-00841-3 34461955 PMC8404342

[B34] Dixit A. Parnas O. Li B. Chen J. Fulco C. P. Jerby-Arnon L. (2016). Perturb-Seq: Dissecting molecular circuits with scalable single-cell RNA profiling of pooled genetic screens. Cell. 167, 1853–1866. e17. 10.1016/j.cell.2016.11.038 27984732 PMC5181115

[B35] Doench J. G. Fusi N. Sullender M. Hegde M. Vaimberg E. W. Donovan K. F. (2016). Optimized sgRNA design to maximize activity and minimize off-target effects of CRISPR-Cas9. Nat. Biotechnol. 34, 184–191. 10.1038/nbt.3437 26780180 PMC4744125

[B36] Douvaras P. Wang J. Zimmer M. Hanchuk S. O’Bara M. A. Sadiq S. (2014). Efficient generation of myelinating oligodendrocytes from primary progressive multiple sclerosis patients by induced pluripotent stem cells. Stem Cell. Rep. 3, 250–259. 10.1016/j.stemcr.2014.06.012 PMC4176529 25254339

[B37] Dräger N. M. Sattler S. M. Huang C. T.-L. Teter O. M. Leng K. Hashemi S. H. (2022). A CRISPRi/a platform in human iPSC-derived microglia uncovers regulators of disease states. Nat. Neurosci. 25, 1149–1162. 10.1038/s41593-022-01131-4 35953545 PMC9448678

[B38] Du Z.-W. Chen H. Liu H. Lu J. Qian K. Huang C.-L. (2015). Generation and expansion of highly pure motor neuron progenitors from human pluripotent stem cells. Nat. Commun. 6, 6626. 10.1038/ncomms7626 25806427 PMC4375778

[B39] Dukhovny A. Lamkiewicz K. Chen Q. Fricke M. Jabrane-Ferrat N. Marz M. (2019). A CRISPR activation screen identifies genes that protect against Zika virus infection. J. Virol. 93, e00211–e00219. 10.1128/JVI.00211-19 31142663 PMC6675891

[B40] Eichmüller O. L. Knoblich J. A. (2022). Human cerebral organoids — A new tool for clinical neurology research. Nat. Rev. Neurol. 18, 661–680. 10.1038/s41582-022-00723-9 36253568 PMC9576133

[B41] Erard N. Knott S. R. V. Hannon G. J. (2017). A CRISPR resource for individual, combinatorial, or multiplexed gene knockout. Mol. Cell. 67, 348–354. e4. 10.1016/j.molcel.2017.06.030 28732207 PMC5526787

[B42] Erwood S. Bily T. M. I. Lequyer J. Yan J. Gulati N. Brewer R. A. (2022). Saturation variant interpretation using CRISPR prime editing. Nat. Biotechnol. 40, 885–895. 10.1038/s41587-021-01201-1 35190686

[B43] Esk C. Lindenhofer D. Haendeler S. Wester R. A. Pflug F. Schroeder B. (2020). A human tissue screen identifies a regulator of ER secretion as a brain-size determinant. Science 370, 935–941. 10.1126/science.abb5390 33122427

[B44] Feldman D. Singh A. Schmid-Burgk J. L. Carlson R. J. Mezger A. Garrity A. J. (2019). Optical pooled screens in human cells. Cell. 179, 787–799. e17. 10.1016/j.cell.2019.09.016 31626775 PMC6886477

[B45] Fleck J. S. Jansen S. M. J. Wollny D. Zenk F. Seimiya M. Jain A. (2022). Inferring and perturbing cell fate regulomes in human brain organoids. Nature, 1–8. 10.1038/s41586-022-05279-8 PMC10499607 36198796

[B46] Flitsch L. J. Laupman K. E. Brüstle O. (2020). Transcription factor-based fate specification and forward programming for neural regeneration. Front. Cell. Neurosci. 14, 121. 10.3389/fncel.2020.00121 32508594 PMC7251072

[B47] Ford K. McDonald D. Mali P. (2019). Functional genomics via CRISPR–cas. J. Mol. Biol. 431, 48–65. 10.1016/j.jmb.2018.06.034 29959923 PMC6309720

[B48] Franklin H. Clarke B. E. Patani R. (2021). Astrocytes and microglia in neurodegenerative diseases: Lessons from human *in vitro* models. Prog. Neurobiol. 200, 101973. 10.1016/j.pneurobio.2020.101973 33309801 PMC8052192

[B49] Freibaum B. D. Taylor J. P. (2017). The role of dipeptide repeats in C9ORF72-related ALS-FTD. Front. Mol. Neurosci. 10, 35. 10.3389/fnmol.2017.00035 28243191 PMC5303742

[B50] Fujimori K. Ishikawa M. Otomo A. Atsuta N. Nakamura R. Akiyama T. (2018). Modeling sporadic ALS in iPSC-derived motor neurons identifies a potential therapeutic agent. Nat. Med. 24, 1579–1589. 10.1038/s41591-018-0140-5 30127392

[B51] Funato H. Miyoshi C. Fujiyama T. Kanda T. Sato M. Wang Z. (2016). Forward-genetics analysis of sleep in randomly mutagenized mice. Nature 539, 378–383. 10.1038/nature20142 27806374 PMC6076225

[B52] Funk L. Su K.-C. Ly J. Feldman D. Singh A. Moodie B. (2022). The phenotypic landscape of essential human genes. Cell. 185, 4634–4653. e22. 10.1016/j.cell.2022.10.017 36347254 PMC10482496

[B53] Garcez P. P. Loiola E. C. Madeiro da Costa R. Higa L. M. Trindade P. Delvecchio R. (2016). Zika virus impairs growth in human neurospheres and brain organoids. Science 352, 816–818. 10.1126/science.aaf6116 27064148

[B54] García-León J. A. García-Díaz B. Eggermont K. Cáceres-Palomo L. Neyrinck K. Madeiro da Costa R. (2020). Generation of oligodendrocytes and establishment of an all-human myelinating platform from human pluripotent stem cells. Nat. Protoc. 15, 3716–3744. 10.1038/s41596-020-0395-4 33097924

[B55] Garland E. F. Hartnell I. J. Boche D. (2022). Microglia and astrocyte function and communication: What do we know in humans? Front. Neurosci. 16, 824888. 10.3389/fnins.2022.824888 35250459 PMC8888691

[B56] Gaudelli N. M. Komor A. C. Rees H. A. Packer M. S. Badran A. H. Bryson D. I. (2017). Programmable base editing of A•T to G•C in genomic DNA without DNA cleavage. Nature 551, 464–471. 10.1038/nature24644 29160308 PMC5726555

[B57] Geirsdottir L. David E. Keren-Shaul H. Weiner A. Bohlen S. C. Neuber J. (2019). Cross-species single-cell analysis reveals divergence of the primate microglia Program. Cell. 179, 1609–1622. e16. 10.1016/j.cell.2019.11.010 31835035

[B58] Gilbert L. A. Horlbeck M. A. Adamson B. Villalta J. E. Chen Y. Whitehead E. H. (2014). Genome-scale CRISPR-mediated control of gene repression and activation. Cell. 159, 647–661. 10.1016/j.cell.2014.09.029 25307932 PMC4253859

[B59] Gönczy P. Echeverri C. Oegema K. Coulson A. Jones S. J. Copley R. R. (2000). Functional genomic analysis of cell division in *C. elegans* using RNAi of genes on chromosome III. Nature 408, 331–336. 10.1038/35042526 11099034

[B60] Guo W. Wang H. Kumar Tharkeshwar A. Couthouis J. Braems E. Masrori P. (2022). CRISPR/Cas9 screen in human iPSC-derived cortical neurons identifies NEK6 as a novel disease modifier of C9orf72 poly(PR) toxicity. Alzheimers Dement. 10.1002/alz.12760 PMC9943798 35993441

[B61] Guttenplan K. A. Stafford B. K. El-Danaf R. N. Adler D. I. Münch A. E. Weigel M. K. (2020a). Neurotoxic reactive astrocytes drive neuronal death after retinal injury. Cell. Rep. 31, 107776. 10.1016/j.celrep.2020.107776 32579912 PMC8091906

[B62] Guttenplan K. A. Weigel M. K. Adler D. I. Couthouis J. Liddelow S. A. Gitler A. D. (2020b). Knockout of reactive astrocyte activating factors slows disease progression in an ALS mouse model. Nat. Commun. 11, 3753. 10.1038/s41467-020-17514-9 32719333 PMC7385161

[B63] Harrison P. J. (2015). Recent genetic findings in schizophrenia and their therapeutic relevance. J. Psychopharmacol. Oxf. Engl. 29, 85–96. 10.1177/0269881114553647 PMC4361495 25315827

[B64] Hart T. Chandrashekhar M. Aregger M. Steinhart Z. Brown K. R. MacLeod G. (2015). High-resolution CRISPR screens reveal fitness genes and genotype-specific cancer liabilities. Cell. 163, 1515–1526. 10.1016/j.cell.2015.11.015 26627737

[B65] Hart T. Tong A. H. Y. Chan K. Van Leeuwen J. Seetharaman A. Aregger M. (2017). Evaluation and design of genome-wide CRISPR/SpCas9 knockout screens. G3 GenesGenomesGenetics 7, 2719–2727. 10.1534/g3.117.041277 PMC5555476 28655737

[B66] Henkel L. Rauscher B. Schmitt B. Winter J. Boutros M. (2020). Genome-scale CRISPR screening at high sensitivity with an empirically designed sgRNA library. BMC Biol. 18, 174. 10.1186/s12915-020-00905-1 33228647 PMC7686728

[B67] Hoffmann H.-H. Schneider W. M. Rozen-Gagnon K. Miles L. A. Schuster F. Razooky B. (2021). TMEM41B is a pan-flavivirus host factor. Cell. 184, 133–148. e20. 10.1016/j.cell.2020.12.005 33338421 PMC7954666

[B68] Hotta Y. Benzer S. (1972). Mapping of Behaviour in Drosophila mosaics. Nature 240, 527–535. 10.1038/240527a0 4568399

[B69] Hulme A. J. Maksour S. St-Clair Glover M. Miellet S. Dottori M. (2021). Making neurons, made easy: The use of Neurogenin-2 in neuronal differentiation. Stem Cell. Rep. 17, 14–34. 10.1016/j.stemcr.2021.11.015 PMC8758946 34971564

[B70] Ihry R. J. Salick M. R. Ho D. J. Sondey M. Kommineni S. Paula S. (2019). Genome-scale CRISPR screens identify human pluripotency-specific genes. Cell. Rep. 27, 616–630. e6. 10.1016/j.celrep.2019.03.043 30970262

[B71] Ihry R. J. Worringer K. A. Salick M. R. Frias E. Ho D. Theriault K. (2018). p53 inhibits CRISPR–Cas9 engineering in human pluripotent stem cells. Nat. Med. 24, 939–946. 10.1038/s41591-018-0050-6 29892062

[B72] Jaitin D. A. Weiner A. Yofe I. Lara-Astiaso D. Keren-Shaul H. David E. (2016). Dissecting immune circuits by linking CRISPR-pooled screens with single-cell RNA-seq. Cell. 167, 1883–1896. e15. 10.1016/j.cell.2016.11.039 27984734

[B73] Jayaraman D. Bae B.-I. Walsh C. A. (2018). The genetics of primary microcephaly. Annu. Rev. Genomics Hum. Genet. 19, 177–200. 10.1146/annurev-genom-083117-021441 29799801

[B74] Jin X. Simmons S. K. Guo A. Shetty A. S. Ko M. Nguyen L. (2020). *In vivo* Perturb-Seq reveals neuronal and glial abnormalities associated with autism risk genes. Science 370, eaaz6063. 10.1126/science.aaz6063 33243861 PMC7985844

[B75] Kampmann M. (2020). CRISPR-based functional genomics for neurological disease. Nat. Rev. Neurol. 16, 465–480. 10.1038/s41582-020-0373-z 32641861 PMC7484261

[B76] Kampmann M. (2018). CRISPRi and CRISPRa screens in mammalian cells for precision biology and medicine. ACS Chem. Biol. 13, 406–416. 10.1021/acschembio.7b00657 29035510 PMC5886776

[B77] Kanfer G. Sarraf S. A. Maman Y. Baldwin H. Dominguez-Martin E. Johnson K. R. (2021). Image-based pooled whole-genome CRISPRi screening for subcellular phenotypes. J. Cell. Biol. 220, e202006180. 10.1083/jcb.202006180 33464298 PMC7816647

[B78] Kantor A. McClements M. E. MacLaren R. E. (2020). CRISPR-Cas9 DNA base-editing and prime-editing. Int. J. Mol. Sci. 21, 6240. 10.3390/ijms21176240 32872311 PMC7503568

[B79] Kim E. Hart T. (2021). Improved analysis of CRISPR fitness screens and reduced off-target effects with the BAGEL2 gene essentiality classifier. Genome Med. 13, 2. 10.1186/s13073-020-00809-3 33407829 PMC7789424

[B80] Koike-Yusa H. Li Y. Tan E.-P. Velasco-Herrera M. D. C. Yusa K. (2014). Genome-wide recessive genetic screening in mammalian cells with a lentiviral CRISPR-guide RNA library. Nat. Biotechnol. 32, 267–273. 10.1038/nbt.2800 24535568

[B81] Kramer N. J. Haney M. S. Morgens D. W. Jovičić A. Couthouis J. Li A. (2018). CRISPR–Cas9 screens in human cells and primary neurons identify modifiers of C9ORF72 dipeptide-repeat-protein toxicity. Nat. Genet. 50, 603–612. 10.1038/s41588-018-0070-7 29507424 PMC5893388

[B82] Krencik R. Zhang S.-C. (2011). Directed differentiation of functional astroglial subtypes from human pluripotent stem cells. Nat. Protoc. 6, 1710–1717. 10.1038/nprot.2011.405 22011653 PMC3198813

[B83] Kuhn M. Santinha A. J. Platt R. J. (2021). Moving from *in vitro* to *in vivo* CRISPR screens. Gene Genome Ed. 2, 100008. 10.1016/j.ggedit.2021.100008

[B84] Labeau A. Simon-Loriere E. Hafirassou M.-L. Bonnet-Madin L. Tessier S. Zamborlini A. (2020). A genome-wide CRISPR-cas9 screen identifies the dolichol-phosphate mannose synthase complex as a host dependency factor for dengue virus infection. J. Virol. 94, e017511–19. 10.1128/JVI.01751-19 PMC7081898 31915280

[B85] Lai M.-C. Lombardo M. V. Baron-Cohen S. (2014). Autism. Lancet 383, 896–910. 10.1016/S0140-6736(13)61539-1 24074734

[B86] Lalli M. A. Avey D. Dougherty J. D. Milbrandt J. Mitra R. D. (2020). High-throughput single-cell functional elucidation of neurodevelopmental disease–associated genes reveals convergent mechanisms altering neuronal differentiation. Genome Res. 30, 1317–1331. 10.1101/gr.262295.120 32887689 PMC7545139

[B87] Lancaster M. A. Knoblich J. A. (2014). Generation of cerebral organoids from human pluripotent stem cells. Nat. Protoc. 9, 2329–2340. 10.1038/nprot.2014.158 25188634 PMC4160653

[B88] Lancaster M. A. Renner M. Martin C.-A. Wenzel D. Bicknell L. S. Hurles M. E. (2013). Cerebral organoids model human brain development and microcephaly. Nature 501, 373, 379. 10.1038/nature12517 23995685 PMC3817409

[B89] Leng K. Rose I. V. L. Kim H. Xia W. Romero-Fernandez W. Rooney B. (2022). CRISPRi screens in human iPSC-derived astrocytes elucidate regulators of distinct inflammatory reactive states. Nat. Neurosci. 25, 1528–1542. 10.1038/s41593-022-01180-9 36303069 PMC9633461

[B90] Li C. Fleck J. S. Martins-Costa C. Burkard T. R. Stuempflen M. Vertesy Á. (2022). Single-cell brain organoid screening identifies developmental defects in autism. 2022.09.15.508118. 10.1101/2022.09.15.508118 PMC10499611 37704762

[B91] Li X. Tao Y. Bradley R. Du Z. Tao Y. Kong L. (2018). Fast generation of functional subtype astrocytes from human pluripotent stem cells. Stem Cell. Rep. 11, 998–1008. 10.1016/j.stemcr.2018.08.019 PMC6178885 30269954

[B92] Li Y. Muffat J. Omer A. Bosch I. Lancaster M. A. Sur M. (2017). Induction of expansion and folding in human cerebral organoids. Cell. Stem Cell. 20, 385–396. e3. 10.1016/j.stem.2016.11.017 28041895 PMC6461394

[B93] Li Y. Muffat J. Omer Javed A. Keys H. R. Lungjangwa T. Bosch I. (2019). Genome-wide CRISPR screen for Zika virus resistance in human neural cells. Proc. Natl. Acad. Sci. 116, 9527–9532. 10.1073/pnas.1900867116 31019072 PMC6510995

[B94] Liddelow S. A. Guttenplan K. A. Clarke L. E. Bennett F. C. Bohlen C. J. Schirmer L. (2017). Neurotoxic reactive astrocytes are induced by activated microglia. Nature 541, 481–487. 10.1038/nature21029 28099414 PMC5404890

[B95] Lin H.-C. He Z. Ebert S. Schörnig M. Santel M. Nikolova M. T. (2021). NGN2 induces diverse neuron types from human pluripotency. Stem Cell. Rep. 16, 2118–2127. 10.1016/j.stemcr.2021.07.006 PMC8452516 34358451

[B96] Liu S. J. Horlbeck M. A. Cho S. W. Birk H. S. Malatesta M. He D. (2017). CRISPRi-based genome-scale identification of functional long noncoding RNA loci in human cells. Science 355, aah7111. 10.1126/science.aah7111 27980086 PMC5394926

[B97] Liu Y. Yu C. Daley T. P. Wang F. Cao W. S. Bhate S. (2018). CRISPR activation screens systematically identify factors that drive neuronal fate and reprogramming. Cell. Stem Cell. 23, 758–771. e8. 10.1016/j.stem.2018.09.003 30318302 PMC6214761

[B98] Mair B. Tomic J. Masud S. N. Tonge P. Weiss A. Usaj M. (2019). Essential gene profiles for human pluripotent stem cells identify uncharacterized genes and substrate dependencies. Cell. Rep. 27, 599–615. e12. 10.1016/j.celrep.2019.02.041 30970261

[B99] Mandegar M. A. Huebsch N. Frolov E. B. Shin E. Truong A. Olvera M. P. (2016). CRISPR interference efficiently induces specific and reversible gene silencing in human iPSCs. Cell. Stem Cell. 18, 541–553. 10.1016/j.stem.2016.01.022 26971820 PMC4830697

[B100] Marceau C. D. Puschnik A. S. Majzoub K. Ooi Y. S. Brewer S. M. Fuchs G. (2016). Genetic dissection of Flaviviridae host factors through genome-scale CRISPR screens. Nature 535, 159–163. 10.1038/nature18631 27383987 PMC4964798

[B101] Maroof A. M. Keros S. Tyson J. A. Ying S.-W. Ganat Y. M. Merkle F. T. (2013). Directed differentiation and functional maturation of cortical interneurons from human embryonic stem cells. Cell. Stem Cell. 12, 559–572. 10.1016/j.stem.2013.04.008 23642365 PMC3681523

[B102] Marsh A. P. (2019). Molecular mechanisms of proteinopathies across neurodegenerative disease: A review. Neurol. Res. Pract. 1, 35. 10.1186/s42466-019-0039-8 33324900 PMC7650105

[B103] McTague A. Rossignoli G. Ferrini A. Barral S. Kurian M. A. (2021). Genome editing in iPSC-based neural systems: From disease models to future therapeutic strategies. Front. Genome Ed. 3, 630600. 10.3389/fgeed.2021.630600 34713254 PMC8525405

[B104] Meng X. Yao D. Chen X. Kelley K. W. Reis N. Thete M. V. (2022). CRISPR screens in 3D assembloids reveal disease genes associated with human interneuron development. 2022.09.06.506845. 10.1101/2022.09.06.506845

[B105] Michel P. P. Hirsch E. C. Hunot S. (2016). Understanding dopaminergic cell death pathways in Parkinson disease. Neuron 90, 675–691. 10.1016/j.neuron.2016.03.038 27196972

[B106] Michels B. E. Mosa M. H. Streibl B. I. Zhan T. Menche C. Abou-El-Ardat K. (2020). Pooled *in vitro* and *in vivo* CRISPR-cas9 screening identifies tumor suppressors in human colon organoids. Cell. Stem Cell. 26, 782–792. e7. 10.1016/j.stem.2020.04.003 32348727

[B107] Miura Y. Li M.-Y. Birey F. Ikeda K. Revah O. Thete M. V. (2020). Generation of human striatal organoids and cortico-striatal assembloids from human pluripotent stem cells. Nat. Biotechnol. 38, 1421–1430. 10.1038/s41587-020-00763-w 33273741 PMC9042317

[B108] Moffat J. Grueneberg D. A. Yang X. Kim S. Y. Kloepfer A. M. Hinkle G. (2006). A lentiviral RNAi library for human and mouse genes applied to an arrayed viral high-content screen. Cell. 124, 1283–1298. 10.1016/j.cell.2006.01.040 16564017

[B109] Muffat J. Li Y. Omer A. Durbin A. Bosch I. Bakiasi G. (2018). Human induced pluripotent stem cell-derived glial cells and neural progenitors display divergent responses to Zika and dengue infections. Proc. Natl. Acad. Sci. 115, 7117–7122. 10.1073/pnas.1719266115 29915057 PMC6142255

[B110] Muffat J. Li Y. Yuan B. Mitalipova M. Omer A. Corcoran S. (2016). Efficient derivation of microglia-like cells from human pluripotent stem cells. Nat. Med. 22, 1358–1367. 10.1038/nm.4189 27668937 PMC5101156

[B111] Nakamura M. Gao Y. Dominguez A. A. Qi L. S. (2021). CRISPR technologies for precise epigenome editing. Nat. Cell. Biol. 23, 11–22. 10.1038/s41556-020-00620-7 33420494

[B112] Neely G. G. Hess A. Costigan M. Keene A. C. Goulas S. Langeslag M. (2010). A genome-wide Drosophila screen for heat nociception identifies α2δ3 as an evolutionarily conserved pain gene. Cell. 143, 628–638. 10.1016/j.cell.2010.09.047 21074052 PMC3040441

[B113] Nolbrant S. Heuer A. Parmar M. Kirkeby A. (2017). Generation of high-purity human ventral midbrain dopaminergic progenitors for *in vitro* maturation and intracerebral transplantation. Nat. Protoc. 12, 1962–1979. 10.1038/nprot.2017.078 28858290

[B114] Notaras M. Lodhi A. Dündar F. Collier P. Sayles N. M. Tilgner H. (2022). Schizophrenia is defined by cell-specific neuropathology and multiple neurodevelopmental mechanisms in patient-derived cerebral organoids. Mol. Psychiatry 27, 1416–1434. 10.1038/s41380-021-01316-6 34789849 PMC9095467

[B115] Nuñez J. K. Chen J. Pommier G. C. Cogan J. Z. Replogle J. M. Adriaens C. (2021). Genome-wide programmable transcriptional memory by CRISPR-based epigenome editing. Cell. 184, 2503–2519. e17. 10.1016/j.cell.2021.03.025 33838111 PMC8376083

[B116] Ordovás L. Boon R. Pistoni M. Chen Y. Wolfs E. Guo W. (2015). Efficient recombinase-mediated cassette exchange in hPSCs to study the hepatocyte lineage reveals AAVS1 locus-mediated transgene inhibition. Stem Cell. Rep. 5, 918–931. 10.1016/j.stemcr.2015.09.004 PMC4649136 26455413

[B117] Otten A. B. C. Sun B. K. (2020). Research techniques made simple: CRISPR genetic screens. J. Invest. Dermatol. 140, 723–728. e1. 10.1016/j.jid.2020.01.018 32200874 PMC8525197

[B118] Pandya H. Shen M. J. Ichikawa D. M. Sedlock A. B. Choi Y. Johnson K. R. (2017). Differentiation of human and murine induced pluripotent stem cells to microglia-like cells. Nat. Neurosci. 20, 753–759. 10.1038/nn.4534 28253233 PMC5404968

[B119] Papazian D. M. Schwarz T. L. Tempel B. L. Jan Y. N. Jan L. Y. (1987). Cloning of genomic and complementary DNA from shaker, a putative potassium channel gene from Drosophila. Science 237, 749–753. 10.1126/science.2441470 2441470

[B120] Park R. J. Wang T. Koundakjian D. Hultquist J. F. Lamothe-Molina P. Monel B. (2017). A genome-wide CRISPR screen identifies a restricted set of HIV host dependency factors. Nat. Genet. 49, 193–203. 10.1038/ng.3741 27992415 PMC5511375

[B121] Pham M. T. Pollock K. M. Rose M. D. Cary W. A. Stewart H. R. Zhou P. (2018). Generation of human vascularized brain organoids. Neuroreport 29, 588–593. 10.1097/WNR.0000000000001014 29570159 PMC6476536

[B122] Popova G. Soliman S. S. Kim C. N. Keefe M. G. Hennick K. M. Jain S. (2021). Human microglia states are conserved across experimental models and regulate neural stem cell responses in chimeric organoids. Cell. Stem Cell. 28, 2153–2166. e6. 10.1016/j.stem.2021.08.015 34536354 PMC8642295

[B123] Potting C. Crochemore C. Moretti F. Nigsch F. Schmidt I. Manneville C. (2018). Genome-wide CRISPR screen for PARKIN regulators reveals transcriptional repression as a determinant of mitophagy. Proc. Natl. Acad. Sci. 115, E180–E189. 10.1073/pnas.1711023115 29269392 PMC5777035

[B124] Rees H. A. Liu D. R. (2018). Base editing: Precision chemistry on the genome and transcriptome of living cells. Nat. Rev. Genet. 19, 770–788. 10.1038/s41576-018-0059-1 30323312 PMC6535181

[B125] Renton A. E. Majounie E. Waite A. Simón-Sánchez J. Rollinson S. Gibbs J. R. (2011). A hexanucleotide repeat expansion in C9ORF72 is the cause of chromosome 9p21-linked ALS-FTD. Neuron 72, 257–268. 10.1016/j.neuron.2011.09.010 21944779 PMC3200438

[B126] Replogle J. M. Norman T. M. Xu A. Hussmann J. A. Chen J. Cogan J. Z. (2020). Combinatorial single-cell CRISPR screens by direct guide RNA capture and targeted sequencing. Nat. Biotechnol. 38, 954–961. 10.1038/s41587-020-0470-y 32231336 PMC7416462

[B127] Ringel T. Frey N. Ringnalda F. Janjuha S. Cherkaoui S. Butz S. (2020). Genome-scale CRISPR screening in human intestinal organoids identifies drivers of TGF-β resistance. Cell. Stem Cell. 26, 431–440. e8. 10.1016/j.stem.2020.02.007 32142663

[B128] Rousseaux M. W. C. Vázquez-Vélez G. E. Al-Ramahi I. Jeong H.-H. Bajić A. Revelli J.-P. (2018). A druggable genome screen identifies modifiers of α-synuclein levels via a tiered cross-species validation approach. J. Neurosci. 38, 9286–9301. 10.1523/JNEUROSCI.0254-18.2018 30249792 PMC6199406

[B129] Ruetz T. J. Kashiwagi C. M. Morton B. Yeo R. W. Leeman D. S. Morgens D. W. (2021). Vitro and *in vivo* CRISPR-Cas9 screens reveal drivers of aging in neural stem cells of the brain. 2021.11.23.469762. 10.1101/2021.11.23.469762 PMC11525198 39358505

[B130] Sala Frigerio C. Wolfs L. Fattorelli N. Thrupp N. Voytyuk I. Schmidt I. (2019). The major risk factors for Alzheimer’s disease: Age, sex, and genes modulate the microglia response to aβ plaques. Cell. Rep. 27, 1293–1306. e6. 10.1016/j.celrep.2019.03.099 31018141 PMC7340153

[B131] Salter M. W. Stevens B. (2017). Microglia emerge as central players in brain disease. Nat. Med. 23, 1018–1027. 10.1038/nm.4397 28886007

[B132] Sanchez C. G. Acker C. M. Gray A. Varadarajan M. Song C. Cochran N. R. (2021). Genome-wide CRISPR screen identifies protein pathways modulating tau protein levels in neurons. Commun. Biol. 4, 736. 10.1038/s42003-021-02272-1 34127790 PMC8203616

[B133] Schmidt R. Steinhart Z. Layeghi M. Freimer J. W. Bueno R. Nguyen V. Q. (2022). CRISPR activation and interference screens decode stimulation responses in primary human T cells. Science 375, eabj4008. 10.1126/science.abj4008 35113687 PMC9307090

[B134] Shalem O. Sanjana N. E. Hartenian E. Shi X. Scott D. A. Mikkelson T. (2014). Genome-scale CRISPR-Cas9 knockout screening in human cells. Science 343, 84–87. 10.1126/science.1247005 24336571 PMC4089965

[B135] Shi Y. Kirwan P. Livesey F. J. (2012). Directed differentiation of human pluripotent stem cells to cerebral cortex neurons and neural networks. Nat. Protoc. 7, 1836–1846. 10.1038/nprot.2012.116 22976355

[B136] Shifrut E. Carnevale J. Tobin V. Roth T. L. Woo J. M. Bui C. T. (2018). Genome-wide CRISPR screens in primary human T cells reveal key regulators of immune function. Cell. 175, 1958–1971. e15. 10.1016/j.cell.2018.10.024 30449619 PMC6689405

[B137] Sin O. Michels H. Nollen E. A. A. (2014). Genetic screens in *Caenorhabditis elegans* models for neurodegenerative diseases. Biochim. Biophys. Acta BBA - Mol. Basis Dis. 1842, 1951–1959. 10.1016/j.bbadis.2014.01.015 24525026

[B138] Sivakumar S. Qi S. Cheng N. Sathe A. A. Kanchwala M. Kumar A. (2022). TP53 promotes lineage commitment of human embryonic stem cells through ciliogenesis and sonic hedgehog signaling. Cell. Rep. 38, 110395. 10.1016/j.celrep.2022.110395 35172133 PMC8904926

[B139] Sloan S. A. Andersen J. Pașca A. M. Birey F. Pașca S. P. (2018). Generation and assembly of human brain region-specific three-dimensional cultures. Nat. Protoc. 13, 2062–2085. 10.1038/s41596-018-0032-7 30202107 PMC6597009

[B140] Steinhart Z. Pavlovic Z. Chandrashekhar M. Hart T. Wang X. Zhang X. (2017). Genome-wide CRISPR screens reveal a Wnt–FZD5 signaling circuit as a druggable vulnerability of RNF43-mutant pancreatic tumors. Nat. Med. 23, 60–68. 10.1038/nm.4219 27869803

[B141] Sternberg S. H. Redding S. Jinek M. Greene E. C. Doudna J. A. (2014). DNA interrogation by the CRISPR RNA-guided endonuclease Cas9. Nature 507, 62–67. 10.1038/nature13011 24476820 PMC4106473

[B142] Takahashi K. Tanabe K. Ohnuki M. Narita M. Ichisaka T. Tomoda K. (2007). Induction of pluripotent stem cells from adult human fibroblasts by defined factors. Cell. 131, 861–872. 10.1016/j.cell.2007.11.019 18035408

[B143] Tcw J. Wang M. Pimenova A. A. Bowles K. R. Hartley B. J. Lacin E. (2017). An efficient platform for astrocyte differentiation from human induced pluripotent stem cells. Stem Cell. Rep. 9, 600–614. 10.1016/j.stemcr.2017.06.018 PMC5550034 28757165

[B144] Thomson J. A. Itskovitz-Eldor J. Shapiro S. S. Waknitz M. A. Swiergiel J. J. Marshall V. S. (1998). Embryonic stem cell lines derived from human blastocysts. Science 282, 1145–1147. 10.1126/science.282.5391.1145 9804556

[B145] Tian A. Muffat J. Li Y. (2020). Studying human neurodevelopment and diseases using 3D brain organoids. J. Neurosci. 40, 1186–1193. 10.1523/JNEUROSCI.0519-19.2019 32024767 PMC7002141

[B146] Tian R. Abarientos A. Hong J. Hashemi S. H. Yan R. Dräger N. (2021). Genome-wide CRISPRi/a screens in human neurons link lysosomal failure to ferroptosis. Nat. Neurosci. 24, 1020–1034. 10.1038/s41593-021-00862-0 34031600 PMC8254803

[B147] Tian R. Gachechiladze M. A. Ludwig C. H. Laurie M. T. Hong J. Y. Nathaniel D. (2019). CRISPR interference-based platform for multimodal genetic screens in human iPSC-derived neurons. Neuron 104, 239–255. e12. 10.1016/j.neuron.2019.07.014 31422865 PMC6813890

[B148] Tzelepis K. Koike-Yusa H. De Braekeleer E. Li Y. Metzakopian E. Dovey O. M. (2016). A CRISPR dropout screen identifies genetic vulnerabilities and therapeutic targets in acute myeloid leukemia. Cell. Rep. 17, 1193–1205. 10.1016/j.celrep.2016.09.079 27760321 PMC5081405

[B149] van der Poel M. Ulas T. Mizee M. R. Hsiao C.-C. Miedema S. S. M. Helder B. (2019). Transcriptional profiling of human microglia reveals grey–white matter heterogeneity and multiple sclerosis-associated changes. Nat. Commun. 10, 1139. 10.1038/s41467-019-08976-7 30867424 PMC6416318

[B150] Vasile F. Dossi E. Rouach N. (2017). Human astrocytes: Structure and functions in the healthy brain. Brain Struct. Funct. 222, 2017–2029. 10.1007/s00429-017-1383-5 28280934 PMC5504258

[B151] Wang B. Wang M. Zhang W. Xiao T. Chen C.-H. Wu A. (2019). Integrative analysis of pooled CRISPR genetic screens using MAGeCKFlute. Nat. Protoc. 14, 756–780. 10.1038/s41596-018-0113-7 30710114 PMC6862721

[B152] Wang S. Zhang Q. Tiwari S. K. Lichinchi G. Yau E. H. Hui H. (2020). Integrin αvβ5 internalizes Zika virus during neural stem cells infection and provides a promising target for antiviral therapy. Cell. Rep. 30, 969–983. e4. 10.1016/j.celrep.2019.11.020 31956073 PMC7293422

[B153] Wang T. Wei J. J. Sabatini D. M. Lander E. S. (2014). Genetic screens in human cells using the CRISPR-Cas9 system. Science 343, 80–84. 10.1126/science.1246981 24336569 PMC3972032

[B154] Wells M. F. Nemesh J. Ghosh S. Mitchell J. M. Salick M. R. Mello C. J. (2023). Natural variation in gene expression and viral susceptibility revealed by neural progenitor cell villages. Cell. Stem Cell. 30, 312–332. e13. 10.1016/j.stem.2023.01.010 36796362 PMC10581885

[B155] Wertz M. H. Mitchem M. R. Pineda S. S. Hachigian L. J. Lee H. Lau V. (2020). Genome-wide *in vivo* CNS screening identifies genes that modify CNS neuronal survival and mHTT toxicity. Neuron 106, 76–89. e8. 10.1016/j.neuron.2020.01.004 32004439 PMC7181458

[B156] Wheeler E. C. Vu A. Q. Einstein J. M. DiSalvo M. Ahmed N. Van Nostrand E. L. (2020). Pooled CRISPR screens with imaging on microraft arrays reveals stress granule-regulatory factors. Nat. Methods 17, 636–642. 10.1038/s41592-020-0826-8 32393832 PMC7357298

[B157] Willsey H. R. Exner C. R. T. Xu Y. Everitt A. Sun N. Wang B. (2021). Parallel *in vivo* analysis of large-effect autism genes implicates cortical neurogenesis and estrogen in risk and resilience. Neuron 109, 788–804. e8. 10.1016/j.neuron.2021.01.002 33497602 PMC8132462

[B158] Yan X. Stuurman N. Ribeiro S. A. Tanenbaum M. E. Horlbeck M. A. Liem C. R. (2021). High-content imaging-based pooled CRISPR screens in mammalian cells. J. Cell. Biol. 220, e202008158. 10.1083/jcb.202008158 33465779 PMC7821101

[B159] Yang J. Rajan S. S. Friedrich M. J. Lan G. Zou X. Ponstingl H. (2019). Genome-scale CRISPRa screen identifies novel factors for cellular reprogramming. Stem Cell. Rep. 12, 757–771. 10.1016/j.stemcr.2019.02.010 PMC6450436 30905739

[B160] Yin J.-A. Frick L. Scheidmann M. C. Trevisan C. Dhingra A. Spinelli A. (2022). Robust and versatile arrayed libraries for human genome-wide CRISPR activation, deletion and silencing. 2022.05.25.493370. 10.1101/2022.05.25.493370 PMC11754104 39633028

[B161] Zaqout S. Kaindl A. M. (2022). Autosomal recessive primary microcephaly: Not just a small brain. Front. Cell. Dev. Biol. 9, 784700. 10.3389/fcell.2021.784700 35111754 PMC8802810

[B162] Zhang W. Yang S.-L. Yang M. Herrlinger S. Shao Q. Collar J. L. (2019). Modeling microcephaly with cerebral organoids reveals a WDR62–CEP170–KIF2A pathway promoting cilium disassembly in neural progenitors. Nat. Commun. 10, 2612. 10.1038/s41467-019-10497-2 31197141 PMC6565620

[B163] Zhang Y. Pak C. Han Y. Ahlenius H. Zhang Z. Chanda S. (2013). Rapid single-step induction of functional neurons from human pluripotent stem cells. Neuron 78, 785–798. 10.1016/j.neuron.2013.05.029 23764284 PMC3751803

